# Idiopathic Parkinson’s Disease and Schizophrenia: Dilemma in Diagnosis and Treatment of a Case

**Published:** 2019-04

**Authors:** Sneha Sharma, Nitin Aggarwal

**Affiliations:** Lady Hardinge Medical College, New Delhi, India.

**Keywords:** *Parkinson’s Disease*, *Schizophrenia*

## Abstract

The relationship between schizophrenia and idiopathic Parkinson’s disease is still not clear and rare when they coexist. Diagnosing coexisting schizophrenia and idiopathic Parkinson’s disease is a chal-lenge, especially in developing countries due to lack of experts and advance imaging facilities. Treatment options are also limited. A 58- year- old male was admitted due to relapse of psychotic symptoms following non-compliance to antipsychotic medications. The patient was previously diag-nosed with schizophrenia and later developed Parkinson’s disease while non-compliant to antipsy-chotic medications. While the patient was at our center, his detailed history was taken and general physical examination was done to distinguish Parkinson’s disease from anti-psychotic induced ex-trapyramidal symptoms. All the routine investigations were within normal limits. This case report highlights the clinical factors that help make a distinction and help use appropriate drugs to manage schizophrenia with comorbid Parkinson’s disease in developing countries, where lack of precise di-agnostic imaging modalities interferes in making a conclusive diagnosis.

Multiple theories have been suggested about the etiology of schizophrenia. The one which is gaining attention is that schizophrenia may be a progressive neurodegenerative disorder associated with dysfunction of various neurotransmitters in the brain. Dopamine is the major neurotransmitter re-searched in the pathogenesis of schizophrenia, leading to the development of various antipsychotics that primarily target the dopamine pathway (1). 

While schizophrenia is linked to a primarily hyper-dopaminergic state, Parkinson’s disease, on the other hand, is caused by decreased dopamine functioning in the nigrostriatal pathway (2). In fact, treatment with most antipsychotics, mostly dopamine antagonists, leads to parkinsonian side-effects termed as extra pyramidal symptoms. Hence, it does not come as a surprise that only a few case reports mention the coexistence of schizophrenia and idiopathic Parkinson’s disease (3)-(6). 

Post-mortem biopsy used to be the only way to make a definitive diagnosis of coexisting Parkinson’s disease in a patient with schizophrenia, usually labelled as drug-induced parkinsonism ante-mortem. 

 Recent advances in the field of neuroimaging have made possible the distinction between Parkinson’s disease and drug induced parkinsonism, which will allow better diagnosis and management. However, the availability and cost of these resources present a challenge for their use in developing countries where clinical history and presentation continue to be the main pillars in diagnosis. The current case report focuses on the challenges faced in the diagnosis and management of coexisting schizophrenia and idiopathic Parkinson’s disease.

## Case Report

Patient A was a 58-year-old, married male, with low socioeconomic urban background and sixth grade education level. He has not been working since the last nine months and was presented as a di-agnosed case of paranoid schizophrenia according to ICD-10 guidelines, at the age of 28 years. He developed symptoms, such as second person auditory hallucinations, delusion of control, persecution and reference, with fluctuating course and total duration of illness of 30 years.

The patient was on treatment with the second generation antipsychotic, Risperidone, for almost 3-4 years during which all his symptoms were controlled and there were no side-effects. During the non-compliance period of 8-9 months due to full control of symptoms, the patient developed unilateral pin-rolling tremors on the right arm and was referred to a senior neurologist. The tremors gradually progressed to involve the contralateral arm. Meanwhile, the patient also developed changes in gait and posture. He was started on levodopa and carbidopa combination and referred to a psychiatrist for the man-agement of emerging psychotic symptoms and his failure to take medications secondary to delusion of persecution. Prior to the onset of schizophrenia, he had no significant history of medical or psychiatric disorder, or any substance use and no significant family history and personal history.

Mental status examination findings on the day of admission was suggestive of an elderly male, dressed appropriately for his socioeconomic status and season, with poor self-care, uncooperative for examination, verbally abusive, and resisting admission. The patient was muttering to himself and had poor attention span. Psychomotor activity was increased and the patient had irritable mood, speech was clear, loud, and non-goal directed. Thought content was suggestive of delusion of persecution, reference, and control. Higher mental functions could not be elicited due to poor attention span. He had impaired judgement and poor insight into the illness.

Routine investigations did not show any abnormality, and a tentative diagnosis of schizophrenia with Parkinson’s disease was confirmed. Patient’s MRI was suggestive of age-related cerebral atrophy, but other imaging such as DAT-SCAN SPECT, could not be done due to unavailability and economical restrictions.

The patient was started on quetiapine at the low dose of 50 mg initially, which was increased to a dose of 800 mg, and subsequent improvement was observed. Within two weeks of the optimal dosing, biological functions (sleep, appetite, bladder-bowel functions) improved, the patient started maintaining self-care appropriately. His aggression disappeared and he started interacting with family members and greeting visitors. He complained of dryness of mouth for which supportive measures were advised without starting a drug. After consulting with a neurologist, he was maintained on levodopa100 mg and carbidopa 25 mg three times a day, and complete symptomatic improvement of Parkinsonian symptoms was noted.

## Discussion

Rare combination of the two disorders pose diagnostic as well as management difficulties for clinicians (4), (7). Since schizophrenia usually has an early age of onset compared to idiopathic Parkinson’s disease, it becomes a challenging task to differentiate antipsychotic-induced parkinsonism from idiopathic Parkinson’s disease based solely on clinical signs and symptoms. However, certain features such as unilateral onset of tremors with characteristic pin-rolling movements are more in favor of a diagnosis of idiopathic Parkinson’s disease. Since in this case parkinsonian symptoms de-veloped when the patient was off antipsychotics, the symptoms were unlikely to be drug-induced. Also, if a patient on antipsychotics does not improve significantly with measures such as dose re-duction or adding anticholinergic agents, it should raise suspicion of idiopathic Parkinson’s disease (3). Certain case reports have suggested that SPECT using dopamine transporter tracer (DAT) has shown a significant bilateral decrease of striatal dopamine in cases of idiopathic Parkinson’s disease in a patient with chronic schizophrenia (5), (6), (8). However, lack of these advanced imaging facili-ties in developing countries makes it difficult to make a conclusive diagnosis, which affects man-agement.

Management of comorbid schizophrenia with Parkinson's disease poses another significant challenge (9), (10). Since there is an inverse relationship of dopamine in two disorders, treatment of one con-dition carries the potential risk of worsening the other. Hence, to treat psychotic symptoms in a case with idiopathic Parkinson’s disease, drugs with faster dopamine receptor dissociation, such as quetiapine, are preferred (11). Another class of drugs which can be used without theoretical worsening of Parkinson’s symptoms are those with poor dopamine selectivity, such as clozapine (12). However, clozapine is not used as first line drug due to its serious side-effect profile. ECT can also be a viable option in such cases as suggested by some case reports.

Pimavanserin is the first antipsychotic approved by FDA for the treatment of psychotic symptoms in Parkinson's disease due to its inverse agonist and antagonistic action at 5-HT receptors (13), (14). However, the drug has limited availability in developing countries and has not been extensively re-searched. 

The present case improved on quetiapine given for schizophrenia and levodopa and carbidopa com-bination for Parkinson's disease. Yet, the progression and course of these two illnesses and the chal-lenges in management that will crop up subsequently remain to be seen on subsequent follow-ups.

## Limitation:

DAT-SCAN SPECT could not be done due to unavailability and economical restrictions.

## References

[1] Carlsson A, Carlsson ML (2006). A dopaminergic deficit hypothesis of schizophrenia: The path to discovery. Dialogues Clin Neurosci.

[2] Dauer W, Przedborski S (2003). Parkinson’s disease: Mechanisms and models. Neuron.

[3] Grover S, Sahoo S, Goyal M (2017). Schizophrenia with comorbid idiopathic parkinson’s disease: A difficult clinical management scenario. Indian J Psychol Med.

[4] de Jong MH, Zemel D, Van Gool AR (2014). Clinical aspects of comorbid schizophrenia and idio-pathic Parkinson’s disease. Clin Schizophr Relat Psychoses.

[5] Winter C, Juckel G, Plotkin M, Niehaus L, Kupsch A (2006). Paranoid schizophrenia and idiopathic Parkinson’s disease do coexist: A challenge for clinicians. Psychiatry Clin Neurosci.

[6] Gadit A (2011). Schizophrenia and Parkinson’s disease: Challenges in management. BMJ Case Rep.

[7] Lam RW (1993). Chronic schizophrenia and idiopathic Parkinson’s disease. Can J Psychiatry.

[8] Plotkin M, Amthauer H, Klaffke S, Kühn A, Lüdemann L, Arnold G (2005). Combined 123I-FP-CIT and 123I-IBZM SPECT for the diagnosis of parkinsonian syndromes: Study on 72 patients. J Neural Transm (Vienna).

[9] Friedman JH (2011). Managing idiopathic Parkinson’s disease in patients with schizophrenic disor-ders. Parkinsonism Relat Disord.

[10] Lan CC, Su TP, Chen YS, Bai YM (2011). Treatment dilemma in comorbidity of schizophrenia and idiopathic Parkinson’s disease. Gen Hosp Psychiatry.

[11] Menza MMA, Palermo B, Mark M (1999). Quetiapine as an alternative to clozapine in the treatment of dopamimetic psychosis in patients with Parkinson’s disease. Ann Clin Psychiatry.

[12] Ruggieri S, De Pandis MF, Bonamartini A, Vacca L, Stocchi F (1997). Low dose of clozapine in the treatment of dopaminergic psychosis in Parkinson’s disease. Clin Neuropharmacol.

[13] Meltzer HY, Mills R, Revell S, Williams H, Johnson A, Bahr D (2010). Pimavanserin, a seroto-nin(2A) receptor inverse agonist, for the treatment of parkinson’s disease psychosis. Neuropsychopharmacology.

[14] Cummings J, Isaacson S, Mills R, Williams H, Chi-Burris K, Corbett A (2014). Pimavanserin for patients with Parkinson’s disease psychosis: A randomised, placebo-controlled phase 3 trial. Lancet.

